# Supplementary material for: Utilization of social health security scheme among the households of Illam district, Nepal
Source: PLoS One. 2022 May 10;17(5):e0265993. doi: 10.1371/journal.pone.0265993 (PMC9089892; doi:10.1371/journal.pone.0265993)
Supplement: S1 Checklist — (DOCX) [file pone.0265993.s001.docx]

COREQ (COnsolidated criteria for REporting Qualitative research) Checklist

| **Topic** | **Item No.** | **Guide Questions/Description** |
| --- | --- | --- |
| Interviewer/facilitator | 1 | Which author/s conducted the interview or focus group?  Ans: Main Author |
| Credentials | 2 | What were the researcher’s credentials? E.g. PhD, MD,  Ans: Master/Post Graduate |
| Occupation | 3 | What was their occupation at the time of the study?  Ans: Student |
| Gender | 4 | Was the researcher male or female?  Ans: Male |
| Experience and training | 5 | What experience or training did the researcher have?  Ans: Training |
| Relationship established | 6 | Was a relationship established prior to study commencement?  Ans: Yes |
| Participant knowledge of  the interviewer | 7 | What did the participants know about the researcher? e.g. personal  goals, reasons for doing the research  Ans: objective of research was explained to participants |
|  |  |  |
|  |  |  |
| Interviewer characteristics | 8 | What characteristics were reported about the inter viewer/facilitator?  e.g. Bias, assumptions, reasons and interests in the research topic  Ans: Master thesis completion |
|  |  |  |
|  |  |  |
| Methodological orientation and Theory | 9 | What methodological orientation was stated to underpin the study? e.g. grounded theory, discourse analysis, ethnography, phenomenology,  content analysis  Ans: Phenomenology |
|  |  |  |
|  |  |  |
| Sampling | 10 | How were participants selected? e.g. purposive, convenience,  consecutive, snowball  Ans: Purposive sampling |
|  |  |  |
|  |  |  |
| Method of approach | 11 | How were participants approached? e.g. face-to-face, telephone, mail,  Email  Ans: Focus group discussion |
|  |  |  |
|  |  |  |
| Sample size | 12 | How many participants were in the study?  Ans: 10 participants for one FGD |
| Non-participation | 13 | How many people refused to participate or dropped out? Reasons?  Ans: Non |
| Setting of data collection | 14 | Where was the data collected? e.g. home, clinic, workplace  Ans: at home, in enclosed setting |
| Presence of non-  participants | 15 | Was anyone else present besides the participants and researchers?  Ans: No |
|  |  |  |
|  |  |  |
| Description of sample | 16 | What are the important characteristics of the sample? e.g. demographic  data, date  Ans: Participants from insured household whose date of service was into effect, ensuring representation of gender, different castes, religious groups, and the elderly |
|  |  |  |
|  |  |  |
| Interview guide | 17 | Were questions, prompts, guides provided by the authors? Was it pilot  tested?  Ans: Focus group discussion guideline was developed and pilot test was done |
|  |  |  |
| Repeat interviews | 18 | Were repeat inter views carried out? If yes, how many?  Ans: No |
| Audio/visual recording | 19 | Did the research use audio or visual recording to collect the data?  Ans: Audio recording was done |
| Field notes | 20 | Were field notes made during and/or after the inter view or focus group?  Ans: Yes |
| Duration | 21 | What was the duration of the inter views or focus group?  Ans: 30-40 Minutes |
| Data saturation | 22 | Was data saturation discussed?  Ans: Yes |
| Transcripts returned | 23 | Were transcripts returned to participants for comment and/or  Ans: No |
| **Topic** | **Item No.** | **Guide Questions/Description** |
|  |  | correction? |
| Number of data coders | 24 | How many data coders coded the data?  Ans: one, researcher |
| Description of the coding  tree | 25 | Did authors provide a description of the coding tree?  Ans: Yes |
|  |  |  |
| Derivation of themes | 26 | Were themes identified in advance or derived from the data?  Ans: identified in advance |
| Software | 27 | What software, if applicable, was used to manage the data?  Ans: Non, done manually |
| Participant checking | 28 | Did participants provide feedback on the findings?  Ans: No |
| Quotations presented | 29 | Were participant quotations presented to illustrate the themes/findings?  Was each quotation identified? e.g. participant number  Ans: Yes |
|  |  |  |
|  |  |  |
| Data and findings consistent | 30 | Was there consistency between the data presented and the findings?  Ans: Yes |
| Clarity of major themes | 31 | Were major themes clearly presented in the findings?  Ans: Yes |
| Clarity of minor themes | 32 | Is there a description of diverse cases or discussion of minor themes?  Ans: No |
