# Supplementary material for: Utilization of social health security scheme among the households of Illam district, Nepal
Source: PLoS One. 2022 May 10;17(5):e0265993. doi: 10.1371/journal.pone.0265993 (PMC9089892; doi:10.1371/journal.pone.0265993)
Supplement: S1 Appendix — (DOCX) [file pone.0265993.s002.docx]

**Appendix**

**Operational Definition**

1. **Social Health Security (SHS) Scheme:** [1]

- Social Health Security Scheme benefit package is the services and drugs that are available to SHSP members at health facilities under the SHSP.
- These include emergency services, out-patient services, select inpatient services, select diagnostic services and select drugs, in addition to any free services and drugs available at public health facilities through other programs.

1. **Utilization of the SHS scheme:** [2]

- Whenever the beneficiary had used the insurance card to use the benefit package or taken the benefits of social health insurance scheme, it will be considered as utilization of scheme.

1. **Major and Minor Morbidity:** [3]

- **Major Morbidity:** Any illness necessitating an admission of more than 24 hours.
- **Minor Morbidity:** Any morbidity of sudden onset which affected the activities of daily living for more than 24 hours.

**References**

1. Social Health Security Program: Standard Operating Procedure. Kathmandu: Government of Nepal Social Health Security Development Committee; 2016. 1-98.

2. Thakur H. Study of Awareness, Enrollment and Utilization of Rashtriya Swasthya Bima Yojana (National Health Insurance Scheme) in Maharastra, India. Frontier Public Health 2016; 3: 1-13.

3. Philip NE. Minor, major morbidity and utilization of comprehensive health insurance scheme, Kerala: a comprehensive study and un-insured BPL households in Trivandrum. Sree Chitra Turunal Institute for Medical Sciences and Technology 2011; Thesis: 1-48.
