# Supplementary material for: Utilization of social health security scheme among the households of Illam district, Nepal
Source: PLoS One. 2022 May 10;17(5):e0265993. doi: 10.1371/journal.pone.0265993 (PMC9089892; doi:10.1371/journal.pone.0265993)
Supplement: Attachment — Submitted filename: Response to Reviewers.docx [file pone.0265993.s004.docx]

**Author’s response to reviewers**

PONE-D-20-013306

**Title:** “Utilization of social health security scheme among the households of Illam district, Nepal”

**Authors:**

Sanjeeb Shah

Nilambar Jha

Vijay Kumar Khanal

Gyanu Nepal Gurung

Babita Sharma

Mausam Shrestha

**Version:** 1 **Date:** 21 November 2020

**Author’s response to editor’**

The needful correction has been made as per the comment received from the editor.

1. The manuscript has been prepare as per PLOS ONE’s style requirements, including those for the file naming
2. Questionnaire

Clarification is done on the questionnaire used in the study under Data collection section for quantitative study at page no 7, line no 136-139.

“Data was collected by researcher himself using pretested semi-structured questionnaires through face to face interview developed through intensive literature review according to research objective and methodology.”

A copy of questionnaire has been included in both the original language and English, as supporting information

3. The manuscript has been thoroughly edited for the language usage, spelling and grammar.

The name of colleague that edited the manuscript is Babita Sharma, MPH (Officer-Monitoring and Evaluation, Karuna Foundation Nepal), Birendra Sah, MD, FCCP (Associate Professor, Department of Medicine, College of Medicine, Upstate Medical University)

A copy of manuscript showing a change by highlighting them has been uploaded as supporting information file

A clean copy of edited manuscript has been uploaded as manuscript file

4. Caption for the ……

**Author’s response to Reviewers’**

**Reviewer #1**

***The Govt. of Nepal has been implementing a Social Health Insurance program, and this particular research aims to assess utilization patterns of the scheme in one district, Illam, Nepal. While it is critical to conduct such exercise given its importance of being used as inputs for monitoring and revising the scheme design, following observations may be useful in respect to the manuscript in its current form.

The manuscript hardly provides any idea about the background of social security program. How many people are covered, which sections of population are covered, are there targeted mechanism? What is the benefit package offered by the scheme, and is this scheme purely health security or a larger social security scheme? and if so, what aspect of the survey captured in the manuscript? What kind of purchasing mechanism did the insurance scheme implement – did they empanel both public and private hospitals? A reading of the text later suggests that the manuscript deals with social health security and not social security scheme at large. Moreover, further down the text in sections dealing with qualitative results, the authors appear to use the term social health insurance. The manuscript must therefore clearly articulate what type of scheme are they referring to?***

- Thank You for your comment.

The background of social health security program has been provided from page 3, line 55 to page 4, line 81 as:

Nepal remains committed to achieving universal health coverage, as seen in the new constitution, which was promulgated in 2015. [2] Although Nepal has a wide network of health facilities, only two-third of population has easy access to health care. [6] Despite various treatment subsidies like incentives program and pilot insurance scheme, out-of-pocket payment (OOP) is still the main way to finance health care which accounts for 48% of total health expenditure. [7] Thus, there was a felt need for a mechanism in the health financing system to enhance prepayment and risk pooling to minimise financial hardship and impoverishment arising from the use of health care services in Nepal. The government of Nepal introduced social health insurance Program as social health security Program in 2016 by rolling out in three districts Kailali, Baglung and Illam as piloting districts. The Social Health Security Programme (SHSP) is a social protection programme of the Government of Nepal that aims to enable its citizens to access quality health care services without placing a financial burden on them. In 2015 Government of Nepal established a semi-autonomous body, Social Health Security Development Committee which is now known as Health Insurance Board as an implementer of program which follows the structure of centre, province, district and ward. For the yearly membership fee (Premium) of Rs. 2500, an insured family of upto 5 member receives insurance card and an annual coverage of benefit package up to Rs. 50000 health related expenses in both public and private social health insurance empanelled hospitals. The scheme include the benefit package of emergency services, out-patient services, selected inpatient services, selected diagnostic services and selected drugs, in addition to any free services and drugs available at public health facilities through other programmes. The government provides subsidy to poor i.e. for ultra-poor 100%, Poor 75% and vulnerable groups 50% discount in premium. The choice of enrolment is voluntary. This cashless scheme covers the pre-existing condition and has no age limits. [2] The coverage rate is 5% covering 2, 38,344 people among the 15 implemented districts. [17]

***Study design:***

1. ***Why was Illam district selected as against other districts? ON what basis the samples were selected? Is that proportionate to social security coverage? The sample size calculation is utterly confusing, the method is unclear. A sample of 300 was collected based on Inpatient utilization of 38.5% (this is unrealistic given that more than one of the population of the district requires hospitalization in a year), which is usually 4-6% in most LMIC countries?***
   Thank You for the comment.

- The reason for the selection of the Illam district against other districts has been mentioned in page no 6, line no 114-116 as

Government of Nepal initiated the Social Health Security program in 2016 AD and piloted in three districts (Kailali, Baglung and Illam) from which Illam district was selected randomly through lottery method.

- The samples were selected based on their insured status and activation of service i.e. those households were selected who were insured and whose date of service has come into effect/activated. This has been mentioned in page no 6, line no 127-128
- Yes, that is proportionate to social security coverage. For this supporting information has been uploaded as “Sampling Procedure” as in page no 6, line no 123-124
- The method of sample size has been made clear in page no…. and line no…. as

A sample size of 300 was calculated using a formula $n={(z}^{2}pq)/e^{2}$ considering the prevalence of In-patient service utilization of 38.5% from the comparative study done in 2016 by Philip NE. et al. [13] which is done as follow: (page 5, line no 97-109)

z = 1.96 (95.0% Confidence level)

Prevalence of In-patient service utilization (p) = 38.5% [13]

q= 100-38.5 =61.5 %

e (margin error)= 15% of p = 15% of 0.385= 0.05775

Sample size (n) = ${(z}^{2}pq)/e^{2}$

n = 1.96*1.96*0.385*0.615 / (.05775)2

= 273.03

n =273

Non response rate: 10.0%

Total sample size = 300

- Yes, a sample of 300 was collected based on In-patient service utilization of 38.5% which is taken from literature of reference number [13] a study done in India as in page 5, line no 99

2. ***Why choose six months rather than one year as data period. If this is for hospitalization did the survey collect information of self-reports for one year for hospitalization or 15/30 days for OP? The choice of recall period for OP and IP is not spelt out in the manuscript.***

Thank You for the comment.

The study was done for the partial fulfilment of Master in Public Health and we were provided the time frame of six months only. The survey collects information only on whether the sample has utilized the In-patient service (IP) or Out-patient services (OP) and other schemes, not the time period of utilization so recall period of OP and IP is not spelt out in manuscript.

3. ***The method of selection of household was even more bizarre. Not sure what spinning the pen techniques is all about? A reference to this technique must be provided, if such a technique is in vogue or were practiced earlier?***

Thank You for the comment.

Spin the pen technique in perhaps the most common implementation; survey teams select a random starting direction from a central location in the cluster by spinning a pen or bottle. Households lying on this transect from the center to the border of the cluster are counted and one of them is then chosen at random. Proximity selection is then used to select subsequent households as the "next nearest" until the desired sample size is reached. More detail on spin the pen technique is given in literature with reference number [18].

4. ***The qualitative method – no information is available in the text about what sets of variables are part of the questionnaire. Besides socio-economic profile of households, what other information were obtained? Did the tools collect information about different illness, did they record utilization of services by different types of providers, and did the researcher collect information about households’ OOP and if so, what are the key variables involved? None of these information/details are provided in the paper.***

Thank You for the comment.

The social health related knowledge, experience of getting enrolled in social health insurance and experience on utilization of social health insurance services are the sets of variable that were the part of the questionnaire in qualitative method. This has been included in page no 13, line no 226 to page no 16, line no 282.

The tool did not collect the information about different illness, did not record the utilization of service by different types of providers, and nor collect the information about households’ OOP. The key variable involved were the Knowledge, experience on getting enrolled and experience on health insurance service utilization. Please find the find the “focus group discussion guideline” as supporting information for more detail on variable for the qualitative part.

5. ***From qualitative method, it was clear that the research was conducted only among those who were insured, leaving the uninsured out. It is equally possible that those who are eligible for insurance but uncovered for any reasons. And this set of population is expected to be significant, which is left out of the survey.***

Thank You for the comment.

Yes, the uninsured population has been left and it has been mention in limitation of the study. Page no 19, line no 368-371

***Data Analysis:
The data analysis section is presented in a vague manner for any meaningful understanding? Why were binary logistic regression analysis chosen. What is the rationale behind choosing this analysis technique? What does the manuscript wants to convey when it says it carried out thematic analysis underlying qualitative data?***

Thank You for the Comment

Binary logistic regression analysis was chosen because the dependent variable is dichotomous/binary i.e utilization of social health insurance which is dependent variable has binary response of “Yes” and “No”. Further detail on the binary logistic regression is given in reference [19] page 7, line no 157

The manuscript wants to convey that for the analysis of the qualitative data thematic analysis method was used. A thematic analysis is one that looks across all the data to identify the common issues that recur, and identify the main themes that summarise all the views you have collected. This is the most common method for descriptive qualitative study. This approach is useful for analysing responses to open-ended questions which was used in this study as “focus group discussion guidelines” (Attached as Supporting Information). For more detail on thematic analysis kindly refer to reference [21]

***Findings:
- For readers in other parts of the world may not be familiar with words such as Brahmin/Chhetri reflecting ethnicity in Nepal. Perhaps a note on these characteristics is required.
- It is unrealistic to expect nearly half of the population (46.7%) to suffer from chronic illness. Either the method adopted to capture illness (self-reports) or the sample technique adopted was skewed. Even assuming nearly half the population have chronic illness in a particular district, does that reflect national average, or global evidence?
- While chronic illness were present in 46.7% of households as reported in Table 1, but
also, it appears that about 10.4% are reported to be hospitalized, which looks extremely high.
- How did the survey go about classifying the disease conditions (broader disease classification) as given in Table 2. Is that based on ICD-10 or what kind of classification?
- The section on qualitative data dealing with social health insurance related knowledge, it is abundantly clear that the scheme is yet to be implemented (since the poverty card in Illam district has not been distributed yet). In the absence of implementation of the scheme in the sample district, it is unclear what kind of results can be expected from the survey? But the next paragraph provides a contradictory view of beneficiaries receiving insurance benefits.***

Thank You for the Comment

- Brahmin/Chhetri reflects upper caste people in society which is noted in page no 9, line 179 as “*Upper caste people in society”
- Nearly half of the population (46.7%) suffered from chronic illness. This might be so because the scheme is voluntary and covers pre-existing conditions too. This led to consumer side moral hazard i.e. those household which has pre-existing condition of chronic illness were more likely to get enrolled in scheme which is significant in this study. A study from India has also shown that the insured households had more number of people with chronic diseases, suggesting adverse selection from the point of view of an insurer, an expected phenomenon in a voluntary health insurance scheme. [13] Moreover going through the national data on chronic illness, according to Department of Health Services annual report 2017/2018 the deaths due to NCDs (cardiovascular disease, diabetes, cancer and respiratory disease) have increased from 60% of all deaths in 2014 to 66% in 2018 (WHO Nepal Country profile 2018).
- This study shows 10.4% of hospitalization (In-patient service utilization) which is not extremely high when comparing with others findings. A study from Kenya. India and China reported 20%, 38.5% 87.4% inpatient service utilization. [12, 13, 14]
- The question regarding the disease condition was open ended and later it was converted in closed end by classifying the disease based on the body system e.g Pneumonia to respiratory system, Hypertension cardiovascular system.
- “Most of them were unaware about subsidize provided by social health insurance board for poor, ultra-poor and marginalized people. This is because health insurance board was unable to implement the policy as government has not distributed the poverty card in Illam district” Here this does not mean that scheme is yet to be implemented, what it means is the one of the policy that government has released regarding the subsidy to be provided on insurance premium for the Poor, ultra-poor and vulnerable group based on poverty card is not provides as government has not distributed the poverty card. It does not mean to say that scheme/social health insurance program is not implementing in illam district.

**Reviewer #2**

***1. The insurance scheme details have not been provided – what are its stated objectives, what kind of people are eligible for insurance scheme, who pays the premium, what part of the population is eligible for waivers, what is the role of government, what kind of providers are used – public, private or both, what kind of care is included – inpatient/out-patient/both etc. If it is a voluntary insurance scheme, it should be mentioned upfront and compared with literature on voluntary insurance.***

The insurance scheme details have been included in page no 3, line no 63-80 as

The government of Nepal introduced social health insurance Program as social health security Program in 2016 by rolling out in three districts Kailali, Baglung and Illam as piloting districts. The Social Health Security Programme (SHSP) is a social protection programme of the Government of Nepal that aims to enable its citizens to access quality health care services without placing a financial burden on them. In 2015 Government of Nepal established a semi-autonomous body, Social Health Security Development Committee which is now known as Health Insurance Board as an implementer of program which follows the structure of centre, province, district and ward. For the yearly membership fee (Premium) of Rs. 2500, an insured family of upto 5 member receives insurance card and an annual coverage of benefit package up to Rs. 50000 health related expenses in both public and private social health insurance empanelled hospitals. The scheme include the benefit package of emergency services, out-patient services, selected inpatient services, selected diagnostic services and selected drugs, in addition to any free services and drugs available at public health facilities through other programmes. The government provides subsidy to poor i.e. for ultra-poor 100%, Poor 75% and vulnerable groups 50% discount in premium. The choice of enrolment is voluntary. This cashless scheme covers the pre-existing condition and has no age limits. [2]

***2. Any literature available on functioning of the scheme and its origin should be included.***

The literature on functioning of scheme and its origin is included as reference [2]

(Social Health Security Program: Standard Operating Procedure. Kathmandu: Government of Nepal Social Health Security Development Committee; 2016. 1-98.)

***3. It is not clear why the scheme was introduced. Though the introduction mentions that the context was of moving away from public sector provisioning, it is not backed by any references.***
The reason behind introducing the scheme has been explained in page no 3, line no 55-63 as

Nepal remains committed to achieving universal health coverage, as seen in the new Constitution, which was promulgated in 2015. [2] Although Nepal has a wide network of health facilities, only two-third of population has easy access to health care. [6] Despite various treatment subsidies like incentives program and pilot insurance scheme, out-of-pocket payment (OOP) is still the main way to finance health care which accounts for 48% of total health expenditure. [7] Thus, there was a felt need for a mechanism in the health financing system to enhance prepayment and risk pooling to minimise financial hardship and impoverishment arising from the use of health care services in Nepal.

***4. The main aspect the study is able to look at is utilisation of insurance scheme by those who were insured. It seems that the families had to bear the premium. It is not surprising that most of those who bought the insurance, used it when they needed it. Socio-economic status of families/individuals has not been captured adequately. Only variable related to that aspect included in the study is of Ethnicity. But it has not been discussed what the results signify.
An example of a significant finding is that utilisation was greater for women than men. The discussion needs to focus on such findings.
The study does not shed much light on many crucial aspects Nepal’s insurance programme – who were more likely to enroll than others, what kind of facilities were accessed and by whom, how did utilisation vary across type of diseases/conditions, what was the difference in out of pocket spending etc. A limitations section is needed.***
The socio-economic status of families is included in findings table 1 as Poverty line of household while those variable with p-value <0.2 in bivariate analysis were only included for the binary logistic regression analysis. Therefore, Poverty line of household was not included for the binary logistic regression as its p-value was 0.253 and not a significant variable for the utilization of scheme. (page 9, line 176-179)

The study has made discussion on the significant finding from Page no 17, line 316 to page no 18, line no 338 as

On the contrary to the study from Kerala India, in this study household having under five children were more likely to utilize the social health security scheme with odds of 2.915 (1.324-6.419) than household not having under five children. [13]

The study suggests that households having chronic illness were 3.830 (1.422-10.316) times more likely to utilize social health security scheme. This finding is consistent with study done in India which showed that presence of chronic illness in family was significantly, 2.1 times more likely to use the health insurance. [13] This suggests an expected phenomenon of voluntary health insurance scheme that there is likely to be adverse selection from an insurer side.

Utilization of social health security scheme was statistically significant with the sex of household members in this study. Males were 0.563 (0.448-0.707) times less likely to utilize the social health security scheme than females. A discussion paper from three African countries presented that Senegal has similar finding to this study i.e. males were less likely to use the health insurance service than females but in other two countries South Africa and Kenya; sex made no difference on service utilization. [12] A study conducted in India showed sex has no role in utilization of health insurance service. [13]

Older age group i.e. 60 and above years of people were significantly more likely to use the social health security scheme than other age group. This finding has been supported by the study from China and South Africa where older population were more likely to use the health service through insurance than other age group. [14, 12] An opposite situation was found in Kenya and Senegal, there was no significant difference in use of health service by senior and non-senior population. [12]

Limitation section has been included in page no 19, line no 368-371. as

The study could not cover the uninsured population. Therefore who were more likely to enrol than others, reason or barrier for not enrolling, what kind of facilities accessed by whom or utilization of service by different provider, what is the difference in out of pocket spending remained uncovered. However, this can be examined in further studies.

***5.The main finding in the qualitative analysis is the discrimination by hospitals between the insured and non-insured. Another important finding is that the poor were not yet given the waiver on premiums. The authors should discuss these aspects in relation to experience in other schemes in various countries.***
The main finding of qualitative analysis has been discussed in page no 19, line no 361-366 as- the qualitative findings (FGD) from the study done in India shared that Rashtriya Swasthya Bima Yojana (National Health Insurance Scheme) the people were unaware about the what services are provided by the Scheme and from which hospital they can get the service. Moreover, insurance did not reached the poor people and discrimination was done based on political power and insurer were compel to make additional payment and pressurized to buy medicine from out of pocket. [9]

***6. The discussion compares figures found in the study with other countries without bothering to check whether the schemes are comparable at all. It is not very useful in its present form. Any comparisons should with schemes with similar design.***

The researcher has tried to compare the figures found in the study with comparable studies from various countries.

***7.The manuscript should mention that this was a descriptive study and no comparison was done.***

The manuscript has mentioned that this is a descriptive study and no comparison was done in page no 5 and line no 91 as “A descriptive cross-sectional method was adopted with mixed method approach”

***8.The language needs to be improved throughout the manuscript.***

Language has been improved with language check by colleagues.
