# Supplementary material for: Utilization of social health security scheme among the households of Illam district, Nepal
Source: PLoS One. 2022 May 10;17(5):e0265993. doi: 10.1371/journal.pone.0265993 (PMC9089892; doi:10.1371/journal.pone.0265993)
Supplement: Attachment — Submitted filename: Response to Reviewers.docx [file pone.0265993.s005.docx]

**Author’s response to reviewers**

PONE-D-20-013306

**Title:** “Utilization of social health security scheme among the households of Illam district, Nepal”

**Authors:**

Sanjeeb Shah

Nilambar Jha

Vijay Kumar Khanal

Gyanu Nepal Gurung

Babita Sharma

Mausam Shrestha

**Version:** 2 **Date:** 16 October 2021

**Author’s response to editor’**

The needful correction has been made as per the comment received from the editor.

1. The manuscript has been thoroughly edited for the language usage, spelling and grammar.

The name of Person who edited the manuscript is Astha Ramaiya, PhD Scholar

A copy of manuscript showing a change by highlighting them has been uploaded as supporting information file

A clean copy of edited manuscript has been uploaded as manuscript file

2. Translation of Nepalese Currency into dollar has been done at page 3 line 66-68 that is Rs. 2500 to $22 and Rs. 50000 to $441 as per 1 USD= 113.38 NPR on 2019/01/18
3. Regarding explanation of Brahmin/Janajati/Dalit distinction it has been noted in Page 9 line 171-172 as:

Brahim/Chhetri-upper caste group, Janjati and Dalit Lower caste/Disadvantage group in society

4. On study Population, It has been mentioned that subject population consist 100% of people who are enrolled in the health insurance scheme in Page 4 line 85-88 as:

A descriptive cross-sectional method was adopted with mixed method approach to study the 300 households that were enrolled in the health insurance scheme of Illam district of Nepal between September 2018 – February 2019 and whose date of service had come into effect.

5. Regarding confusion on term ” Utilization” explanation of Utilization of scheme has been mentioned in section operational definition, page 20 line 383-386 as:

Whenever the beneficiary had used the insurance card to use the benefit package or taken the benefits of social health insurance scheme, it will be considered as” utilization of scheme”

**Author’s response to Reviewers’**

**Reviewer #1**

***The choice of recall period for OP and IP is not spelt out in the manuscript. The response of the authors is unclear. It appears from the description that it is current utilisation and apparently did not seek information about utilisation in the past – a relatively longer period from hospitalisation and a shorter recall period for outpatient visits. And yet the current utilisation rate is surprisingly large and therefore unreal.***

-The choice of recall period for OP and IP and other schemes is spelt out in the manuscript in Data collection section in page 6, line no 132-134 as:

Information on utilization of In-patient and Out-patient and other schemes was obtained for 12 months before the date of interview.

***Concepts are still not clear. The description of utilisation rate of social health insurance is unclear. What is the numerator and denominator used in the calculation of utilisation rate? Similarly, how are underutilisation measured – please indicate the numerator and denominator.***

- Here Utilization of social health insurance is described in section operational definition, page 20 line 383-386 as:

Whenever the beneficiary had used the insurance card to use the benefit package or taken the benefits of social health insurance scheme, it will be considered as” utilization of scheme”

- The numerator and denominator used in the calculation of utilisation rate are “Household utilized social health insurance” and “Total Sample Size” respectively.

$Utilization rate=\left( Household utilized social health insurance\div Total Sample size \right)\times100$

$=\left( 266\div300 \right)\times100$

= 88.7

- The numerator and denominator used to measure underutilisation are “Household did not utilize social health insurance” and “Total Sample size” respectively.

$Underutilization rate=(Household did not utilize social health insurance\div Total Smaple size)\times100$

$=(34\div300)\times100$

*= 11.3*

***The sample size calculation estimating 300 samples was apparently done based on the prevalence of In-patient service utilisation of 38.5%. It is highly unlikely that one in three insured persons are hospitalised at any given point in time. On the contrary, Table 2 provides information about in-patient utilisation at 10.4%.***

- Yes from literature of reference number [13] a study done in India as in page 5, line no 99, a sample of 300 was collected based on In-patient service utilization of 38.5% which is taken

The reason for high In-patient utilization in the study done in India is that the Comprehensive health insurance scheme covered only Inpatient care which lead to increase in inpatient utilization (Mentioned in article discussion section, Philip NE, Kannan S, Sharma SP. Utilization of Comprehensive Health Insurance Scheme, Kerala: A Comparative Study of Insured and Uninsured Below-Poverty-Line Households. Asia-Pacific Journal of Public Health 2016; 28: 77-85.)

***The response from authors about chronic illness being high needs to be reconfirmed. It is equally likely that chronic disease conditions may predominate in a community and increasingly so. But the manuscript is unclear about whether percentage mentioned for chronic conditions are distribution of chronic conditions among the insured or the rate of chronic conditions? In its current form the manuscript gives an impression that the rate of chronic conditions among the entire population is 47%, implying that half of the insured population have chronic conditions. This again seems unreal and unscientific, highlighting the bias in sample calculation***

The percentage mentioned for chronic condition are distribution of chronic conditions among the insured sampled household (that is out of 300 hundred insured sampled household 140 household had chronic illness 46.7%). It’s not the rate of chronic condition among the entire population.

This might be so because the scheme is voluntary and covers pre-existing conditions too. This led to consumer side moral hazard i.e. those household which has pre-existing condition of chronic illness were more likely to get enrolled in scheme which is significant in this study.

***Finally, the manuscript needs to be edited by a professional editor. In its current form, the manuscript suffers from severe language issues, including grammatical mistakes, improper sentence formation, etc. One can find such instances almost at every paragraph, which requires a thorough overhaul of the language.***

The manuscript was edited by Astha Ramaiya, PhD Scholar to solve the language issues, including grammatical mistakes and improper sentence formation.

**Reviewer #2**

***The authors have not addressed the concerns raised by the reviewer. The design for the quantitative analysis remains inadequate***.

The author have addressed the concern raised by the reviewer which is highlighted in manuscript

The design for the quantitative analysis has been explained in page no. 7 line 142-150 as: Collected data was entered in Microsoft Excel 2010 and was analyzed in Statistical Package for Social Science 11.5. Descriptive, bivariate and multivariate analysis was done. Bivariate analysis was done using chi-square test to establish relationship between dependent (utilization of social health security scheme) and independent (household characteristics) variables. The level of significance was set at 5%. Variables with p-value<0.2 and cell count more than 5 in the bivariate analysis were included for the binary logistic regression analysis to find the factors associated with utilization of social health security scheme. For qualitative data thematic analysis was conducted

Binary logistic regression analysis was chosen because the dependent variable is dichotomous/binary i.e utilization of social health insurance which is dependent variable has binary response of “Yes” and “No”. Further detail on the binary logistic regression is given in reference [19] page 23, line no 443.
