# Supplementary material for: Utilization of social health security scheme among the households of Illam district, Nepal
Source: PLoS One. 2022 May 10;17(5):e0265993. doi: 10.1371/journal.pone.0265993 (PMC9089892; doi:10.1371/journal.pone.0265993)
Supplement: Attachment — Submitted filename: Response to Reviewers.docx [file pone.0265993.s006.docx]

**Author’s response to reviewers**

PONE-D-20-13306R2

**Title:** “Utilization of social health security scheme among the households of Illam district, Nepal”

**Authors:**

Sanjeeb Shah

Nilambar Jha

Vijay Kumar Khanal

Gyanu Nepal Gurung

Babita Sharma

Mausam Shrestha

**Version:** 3 **Date:** 10 March 2022

**Author’s response to editor’**

The needful correction has been made as per the comment received from the editor.

1. A statement in the abstract “A . . . study was conducted among 300 households of Illam district which had utilized the social health security scheme” has been changed at page no. 1 line no 18-20 as “A descriptive cross-sectional mixed-method study was conducted among 300 households of Illam district who had enrolled in the social health security scheme”.
2. In introduction; the service benefit is available only through certain facilities has been explained at page no. 3 line no 59-62 as “the government of Nepal introduced the social health insurance program to government run health facility (Primary Health Care Centre, District Hospital which are reachable within one hours) that provide out-patient and in-patient service to mitigate the financial hardship due to healthcare and increase access to healthcare”.
3. For the power analysis; it has changed as follow at page no. 5 line no 95-97 as “A sample size of 300 was calculated using a formula $n={(z}^{2}pq)/e^{2}$ considering the prevalence from study done in 2016 by Philip NE. et al. in Kerala India and non-response rate of 10%”.
4. Regarding the map generation for “spin the pen”, explanation has been provided at page no. 6 line no. 118-120 as “For this map was generated manually with support of social health insurance supervisor of Illam district based on data available there”.
5. Reporting for qualitative component, based on COREQ checklist information has been provided at page no. 7 line no. 130-139 & 150-151 as “The principle of phenomenology was used to conduct three focus group discussions (FGD) in selected three municipalities (one in each municipality) using pretested focus group guideline prepared based on the theme. Each focus group discussion lasted for 30-40 minutes which included 10 participants chose purposively from insured household, whose date of service was into effect, ensuring representation of gender, different castes, religious group and elderly. Note taking as well as audio recording of discussion was done after taking consent from participants. The purpose of FGD was to record the community’s perception of social health security scheme.

For qualitative, data was coded, transcribed and thematic analysis was performed manually where themes were identified in advance.”

COREQ checklist information has been uploaded as supplementary document too

6. Ethnic categories and family type has been explained in text at page no. 8 line no. 165-169 as Brahmin/Chhetri are considered to be the upper caste group, Janjati and Dalit are considered to be the lower caste/disadvantage group in society and nuclear families comprises of parents and children whereas other households were extended families including other relatives too.

Regarding the eligibility for enrollment in the scheme as a single unit and accessibility of service benefit has been mentioned at page no. 4 line no. 69-72 as “For a yearly membership fee (Premium) of Rs. 2500 ($22), a family of upto five member could receive an insurance card and an annual coverage of benefit package up to Rs. 50000 ($441) health related expenses in both public and private social health insurance empanelled hospitals”

The concept of “minor morbidity” has been defined at page no. 20 line no. 370-371 as “Any morbidity of sudden onset which affected the activities of daily living for more than 24 hours.”

7. The discussion part has been modified as per provided comments at page no. 16-19 line no. 281-353 as

“**Utilization of Social Health Security Scheme**

Limited studies have been conducted on the social health insurance program in the context of Nepal. The study revealed higher utilization of social health security scheme which is consistent with the studies done in Philippines and Rwanda. [8, 11] The utilization of outpatient service was higher than inpatient service which aligned with the finding from South Africa, Kenya, Senegal and contrast to India and China. [12, 13, 14] The underutilization of the scheme was slightly lower than that of study from Philippines. [8] The reason for this might be due to easy access to private health services.

**Factors associated with utilization of social health security scheme**

The study depicted that households having under five children and chronic illness were more likely to utilize the social health security scheme, similar to India’s finding [13] suggesting expected phenomenon of voluntary health insurance scheme that there is likely to be adverse selection from an insurer side. In contrast, to study done in South Africa, Kenya and India sex has made difference on scheme utilization in this study. Regarding age, older age group (i.e. 60 and above years of people were more likely to utilize the social health security scheme which is similar with to finding from South Africa and China. [12, 14]

**Community’s Perception**

The FGD enlightened that people had understood what social health insurance program is all about. They came to know about it through radio, television, FCHVs and enrollment assistant. However, none of them exactly knew about what services are provided through social health insurance in hospital and most of them were unaware about the subsidize provided by social health insurance board for poor, ultra-poor and marginalized people as the insurance board was unable to implement the policy because government has not distributed the poverty card in Illam district.

Irrespective of people different financial status, they had enrolled in social health insurance because they had the concept that health insurance protects their family from unpredicted financial risk when they get health problem. In addition, it was easy for people to get enrolled in social health insurance because for each ward one enrollment assistant has been appointed.

People supported the social health insurance program but they had unpleasant experience while taking service at hospital. They faced discrimination with uninsured people, misbehaviors at hospital pharmacy, have to go through numbers of registration counters, and did not find the doctors, all kind of lab services, drugs, and difficulty with referral system. Therefore, for the further improvement of the social health insurance program, people suggested to treat everyone equally in hospital, all the services should be available in nearby hospital, should be able to use insurance card at any place, at any time, at any hospital under social health insurance without going through referral procedure. The qualitative findings (FGD) from the study done in India shared that Rashtriya Swasthya Bima Yojana (National Health Insurance Scheme) the people were unaware about the what services are provided by the Scheme and from which hospital they can get the service. Moreover, insurance did not reached the poor people and discrimination was done based on political power and insurer were compel to make additional payment and pressurized to buy medicine from out of pocket. [9]

**Limitations**

There might have occurred recall bias as the recall period of utilization of service was 12 month before the interview. However, it was minimized with medical bills and report wherever provided but was not applicable in each household. The study could not cover the uninsured population. Therefore who were more likely to enrol than others, reason or barrier for not enrolling, what kind of facilities accessed by whom or utilization of service by different provider, what is the difference in out of pocket spending remained uncovered. However this can be examined in further studies.

**Conclusion and Recommendation**

In Nepal with increased focus on achieving universal health coverage through increasing access to health care, the study provides intuition to which extent this dimension of universal health coverage has been achieved through social health security scheme program in Illam district. Although the utilization of social health security scheme was higher and had positive response towards the program, people were unsatisfied with the service and facility provided by the hospital under the social health security scheme. Therefore, the investment of demand-side will not be justified unless the basic health care infrastructures are upgraded to specialist health besides serving as gatekeeping for specialist service. In addition to that the concern authority should invest in system to monitor the hospitals and develop a feedback mechanism from users so to evaluate the implementation of health insurance scheme. This will further help the government to refine the policy, plan and strategies in addressing the factors that hindered the consumers’ satisfaction. Moreover, such system will provide robust path in achieving the government’s goal of scaling up the program nationwide. Further, a study from provider perspective would give more clear insight on implementation situation of social health security program.”

8. Translation of Nepalese Currency into dollar has been done at page 3 line 66-68 that is Rs. 2500 to $22 and Rs. 50000 to $441 as per 1 USD= 113.38 NPR on 2019/01/18
9. On study Population, It has been mentioned that subject population consist 100% of people who are enrolled in the health insurance scheme in Page 4 line 85-88 as:

A descriptive cross-sectional method was adopted with mixed method approach to study the 300 households that were enrolled in the health insurance scheme of Illam district of Nepal between September 2018 – February 2019 and whose date of service had come into effect.

10. Regarding confusion on term ” Utilization” explanation of Utilization of scheme has been mentioned in section operational definition, page no.19- 20 line 363-366 as:

Whenever the beneficiary had used the insurance card to use the benefit package or taken the benefits of social health insurance scheme, it will be considered as” utilization of scheme”

11. A copy of manuscript showing a change by highlighting them has been uploaded as supporting information file

A clean copy of edited manuscript has been uploaded as manuscript file

**Author’s response to Reviewers’**

**Reviewer #1**

***The choice of recall period for OP and IP is not spelt out in the manuscript. The response of the authors is unclear. It appears from the description that it is current utilisation and apparently did not seek information about utilisation in the past – a relatively longer period from hospitalisation and a shorter recall period for outpatient visits. And yet the current utilisation rate is surprisingly large and therefore unreal.***

-The choice of recall period for OP and IP and other schemes is spelt out in the manuscript in Data collection section in page 6, line no 127-129 as:

Information on utilization of In-patient and Out-patient and other schemes was obtained for 12 months before the date of interview.

***Concepts are still not clear. The description of utilisation rate of social health insurance is unclear. What is the numerator and denominator used in the calculation of utilisation rate? Similarly, how are underutilisation measured – please indicate the numerator and denominator.***

- Here Utilization of social health insurance is described in section operational definition, page no. 19-20 line no. 363-366 as:

Whenever the beneficiary had used the insurance card to use the benefit package or taken the benefits of social health insurance scheme, it will be considered as” utilization of scheme”

- The numerator and denominator used in the calculation of utilisation rate are “Household utilized social health insurance” and “Total Sample Size” respectively.

$Utilization rate=\left( Household utilized social health insurance\div Total Sample size \right)\times100$

$=\left( 266\div300 \right)\times100$

= 88.7

- The numerator and denominator used to measure underutilisation are “Household did not utilize social health insurance” and “Total Sample size” respectively.

$Underutilization rate=(Household did not utilize social health insurance\div Total Smaple size)\times100$

$=(34\div300)\times100$

*= 11.3*

***The response from authors about chronic illness being high needs to be reconfirmed. It is equally likely that chronic disease conditions may predominate in a community and increasingly so. But the manuscript is unclear about whether percentage mentioned for chronic conditions are distribution of chronic conditions among the insured or the rate of chronic conditions? In its current form the manuscript gives an impression that the rate of chronic conditions among the entire population is 47%, implying that half of the insured population have chronic conditions. This again seems unreal and unscientific, highlighting the bias in sample calculation***

The percentage mentioned for chronic condition are distribution of chronic conditions among the insured sampled household (that is out of 300 hundred insured sampled household 140 household had chronic illness 46.7%). It’s not the rate of chronic condition among the entire population.

This might be so because the scheme is voluntary and covers pre-existing conditions too. This led to consumer side moral hazard i.e. those household which has pre-existing condition of chronic illness were more likely to get enrolled in scheme which is significant in this study.

**Reviewer #2**

***The authors have not addressed the concerns raised by the reviewer. The design for the quantitative analysis remains inadequate***.

The author have addressed the concern raised by the reviewer which is highlighted in manuscript

The design for the quantitative analysis has been explained in page no. 7 line 142-149 as: “Collected data was entered in Microsoft Excel 2010 and was analyzed in Statistical Package for Social Science 11.5. Descriptive, bivariate and multivariate analysis was done. Bivariate analysis was done using chi-square test to establish relationship between dependent (utilization of social health security scheme) and independent (household characteristics) variables. The level of significance was set at 5%. Variables with p-value<0.2 and cell count more than 5 in the bivariate analysis were included for the binary logistic regression analysis to find the factors associated with utilization of social health security scheme”.

Binary logistic regression analysis was chosen because the dependent variable is dichotomous/binary i.e utilization of social health insurance which is dependent variable has binary response of “Yes” and “No”. Further detail on the binary logistic regression is given in reference [19] page 22, line no 423.
